# Breaking Azacalix[4]arenes into Induline Derivatives

**DOI:** 10.3390/molecules28248113

**Published:** 2023-12-15

**Authors:** Zhongrui Chen, Gabriel Canard, Olivier Grauby, Benjamin Mourot, Olivier Siri

**Affiliations:** Centre Interdisciplinaire de Nanoscience de Marseille (CINaM), UMR 7325 CNRS Aix-Marseille Université, Campus de Luminy, Case 913, F-13288 Marseille, France; zhongrui.chen@buct.edu.cn (Z.C.); gabriel.canard@univ-amu.fr (G.C.); olivier.grauby@univ-amu.fr (O.G.); benjamin.mourot@cnrs.fr (B.M.)

**Keywords:** azacalix[4]arenes, macrocycles, phenazinium, nanostructure

## Abstract

Tetraamino-tetranitro-azacalixarene **5** is at the crossroad of two different families of compounds depending on the conditions and the agent used to reduce the NO_2_ groups: (1) azacalixphyrin **7** in neutral medium, or (2) phenazinium of type **8** in acidic medium. The key role of the N-substituted amino functions at the periphery is highlighted by investigating octaaminoazacalixarene as a model compound, and by using the corresponding tetrahydroxy-tetranitro-azacalixarene **15** as a precursor, which behaves differently.

## 1. Introduction

Calix[n]arenes have been the focus of much attention in supramolecular chemistry for decades due to their specific molecular structure, which allows the formation of host–guest complexes [1,2,3]. Considerable effort is now devoted to derivatizing the basic backbones of calixarenes, and the most recent developments concern the preparation of analogues by replacing methylenic bridges with heteroatoms in order to tune and improve their properties [4]. Among the various types of heterocalixarenes, thiacalixarenes incorporating sulfur bridge atoms have been the most extensively studied, as their syntheses can be performed using simple and versatile one-step procedures [5,6,7,8]. The introduction of sulfur in place of CH_2_ bridges has opened up a wide range of possibilities, offering many new features compared with “classical” calixarenes. For intance, thiacalix[4]arenes with one bridge oxidized to sulfoxide (**1**) can react with organolithium compounds to form cleaved linear structures **2** that are otherwise very difficult to access [9].


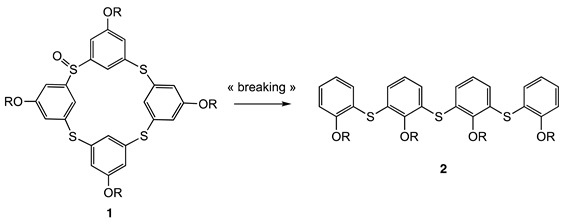


The special functionalities conferred by heteroatoms have stimulated research progress in the field of other heterocalixarenes such as oxacalixarenes [10,11,12,13] and afterwards azacalixarenes, which have been studied in less detail to date. Although the first paper on azacalixarenes **3** was reported in 1963 by Smith (X-ray structure determination) [14], it was not until the late 90s and the reported Buchwald–Hartwig amination (Pd catalyzed) that the synthesis of analogues appeared in the literature [15,16,17,18,19,20,21]. The introduction of nitrogen-bridging atoms on the calix[4]arene scaffold has numerous consequences such as in supramolecular chemistry with assemblies of azacalixarenes [20,21,22]. N-Alkylation of **3** was also a powerful tool to build more sophisticated receptors [23] or introducing inherent chirality [24]. The use of the nitrogen bridge as a spin-bearing site was on the other hand described (upon oxidation) to produce stable radical cations, high-spin diradicals, or polycationic species [25,26,27,28,29,30,31].


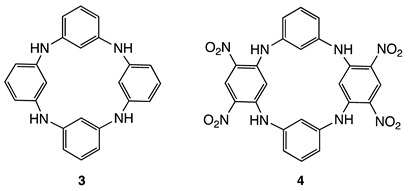


**Figure 1:**
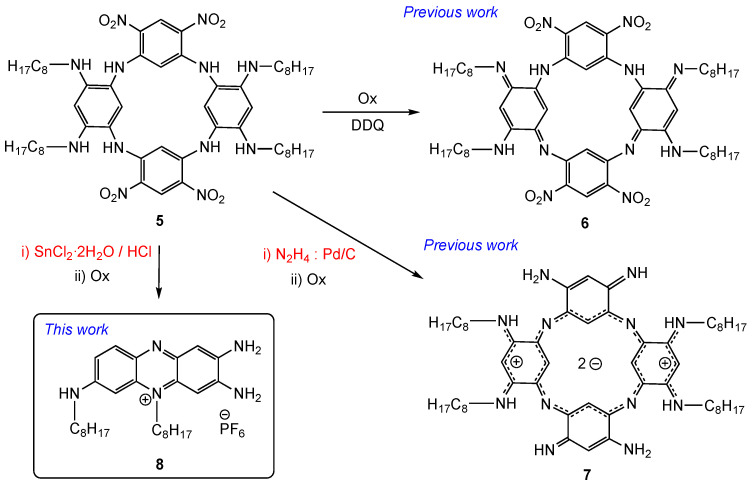
Versatility of tetranitro-tetraaminoazacalixarenes **5**.

At the end of the 2000s, another approach based on aromatic nucleophilic substitutions between a 1,3-diaminobenzene derivative and an electron-poor aryl (typically 1,3-difluoro-4,6-dinitrobenzene) allowed the extending of the library of molecules (**4**) [32,33,34], opening up a wide range of applications [35]. One of the most striking characteristics of tetranitroazacalixarenes **4** is the strong conjugation of the nitrogen lone pair of each bridge with the NO_2_ groups which is responsible for the exclusive 1,3-alternate conformation adopted by these macrocycles so far [36]. This smart and stable conformation produces a close spatial proximity between the appended substituents that were exploited in common recognition processes [34]. Next, the introduction of amino functions at the periphery of tetranitroazacalixarenes (**5**) was considered, as the macrocycle then possesses two tetraaminobenzene-type subunits, well known to be oxidized to the corresponding benzoquinonediimine due to their extremely rich electronic character [37]. This approach led to a new family of macrocycles of type **6**, called azacalixquinarenes, via an oxidation reaction with 2,3-dichloro-5,6-dicyano-1,4-benzoquinone (DDQ) (Figure 1). The resulting molecule consists of two diaminobenzoquinonediimine units linked by dinitrobenzene moieties [37].

Interestingly, the electron-withdrawing nature of the dinitrobenzene moieties in **6** can trigger the intramolecular H-transfer, generating zwitterionic-ground state quinones. The nature of the N-substituents and the polarity of the solvent appear also to have a crucial impact on the equilibrium between the canonical and zwitterionic forms, which exhibit distinct optical and electrochemical properties. Beyond the interest of its oxidation, the reduction reaction of the same molecule **5** appeared also particularly attractive. Tetranitro-tetraaminoazacalixarene **5** alone is indeed remarkably specific as the reduction of its NO_2_ groups with hydrogen on Pd/C leads to the formation of the corresponding, extremely unstable octaaminoazacalixarene (not isolated), which oxidizes in air to form a new class of porphyrin analogs **7** known as azacalixphyrins [38].

Herein, we wish to describe that when the same precursor **5** was instead reduced with SnCl_2_/HCl, the formation of a pink solid, highly emissive in solution, was observed. Spectroscopic investigations demonstrated that azacalixarene **5** is in fact at the crossroad of two different families of dyes depending on the experimental conditions of reduction: the known azacalixphyrins **7** [38] or a triamino-phenazinium **8**. The key role of the amino functions at the periphery of the macrocycle was demonstrated by investigating two analogues with different functions at the periphery.

## 2. Results

**Scheme 1:**
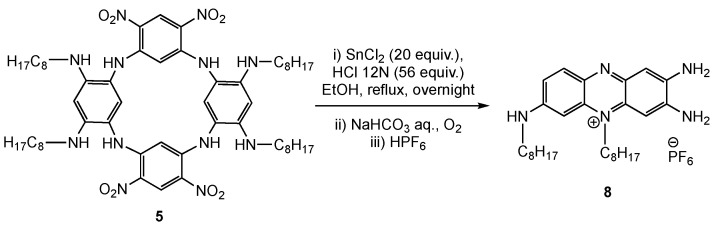
Synthesis of the induline 3B derivative **8**.

**Figure 2:**
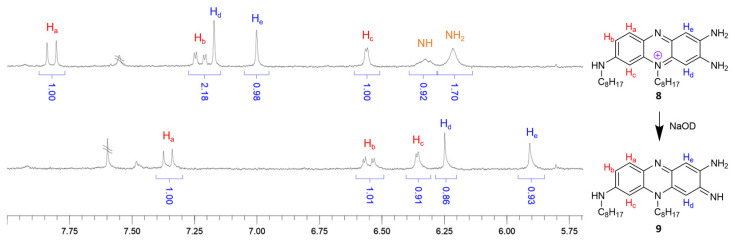
^1^H NMR spectra of **8** (top) and **9** (bottom, after addition of NaOD) in MeCN (the window between δ = 5.7 ppm and 0 ppm has been omitted for clarity).

Compound **5** was prepared as described in the literature [39]. Its reduction was carried out with SnCl_2_∙2H_2_O (32 equiv.) in the presence of HCl (12N) under air (reflux overnight). After neutralization with NaHCO_3_ and anion exchange reaction, **8** was obtained as a dark red solid in a 62% yield (Scheme 1). Mass spectrometry revealed a peak at *m*/*z* = 450.4 for compound **8**, reflecting a “breaking” in the macrocycle (a peak corresponding to *m*/*z* = 923.3 was expected for the macrocycle **7**). The ^1^H NMR spectrum of **8** confirmed this hypothesis, notably with the presence of three aromatic protons showing a doublet signal featuring the substitution pattern indicated in Figure 2 (Ha, Hb, and Hc). Interestingly, the signals of the Hd and He protons in **8** at δ = 7.17 and 7.00 ppm, respectively, undergo a very significant shielding effect (Hd = 6.25 ppm and He = 5.91 ppm) after addition of NaOD (40 wt. % in D_2_O).

This observation is consistent with a quinoidal (i.e., non-aromatic) rearrangement of the molecule [40,41,42]. In other words, molecule **8** is capable, in basic medium, of sacrificing its aromatic character in favor of a non-aromatic species (Figure 2).

**Figure 3:**
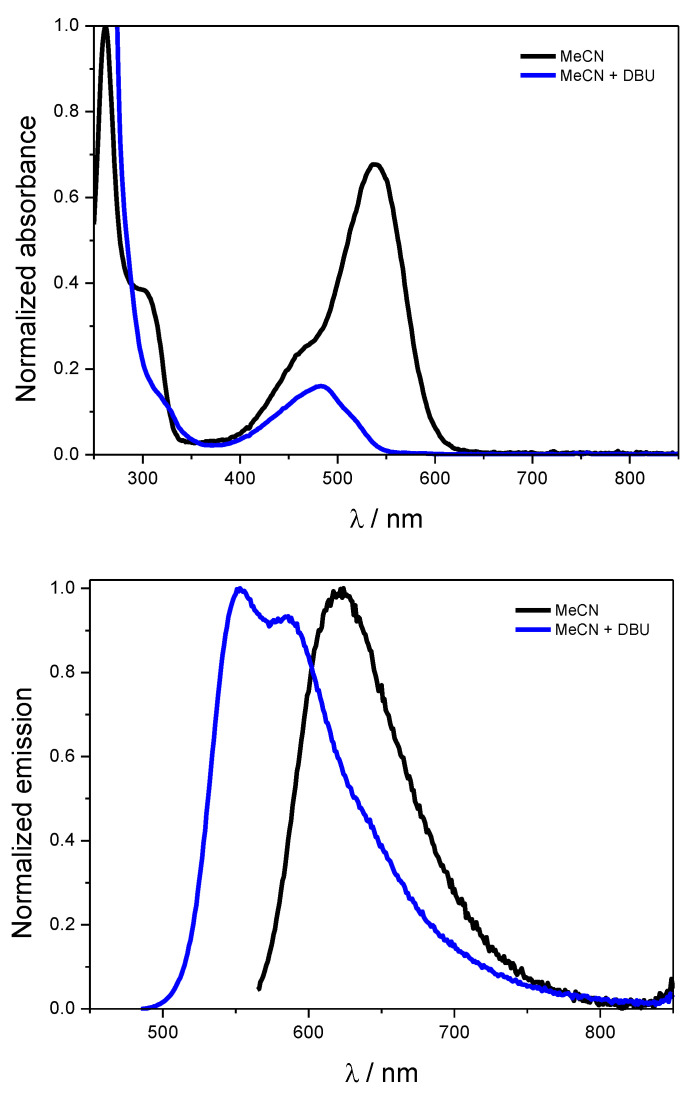
Normalized absorption (**top**) and emission (**bottom**) spectra of **8** (black) and **9** (blue, after addition of DBU) in MeCN.

The UV-Vis absorption spectrum of phenazinium **8** in acetonitrile presents bands in the high energy range and a broad band in the green region covering the 400–600 nm domain and peaking at 538 nm, with a shoulder at λ = 460 nm (Figure 3). In the presence of DBU (up to 4 equiv.), the complete disappearance of **8** is monitored in favor of the formation of neutral quinoidal form **9**, exhibiting a broad blue-shifted absorption in the blue region at λ = 460 nm. Molecule **8** is also emissive (fluorescence) at λ_em_ = 621 nm in MeCN (excitation at λ = 560 nm) (Figure 3). Upon addition of DBU, the fluorescence is blue-shifted to a dual emission at 552 and 585 nm (formation of **9**) and significantly quenched (excitation at λ = 475 nm). These optical data are consistent with similar triamino-phenaziniums recently reported in the literature [41], except for the presence of a dual emission for **9,** due probably to excited-state intramolecular proton transfer [42].

## 3. Discussion

Molecule **8** belongs to the induline 3B family **10**, which has a long history in the field of textile and paint pigments [43,44,45]. Although described in 1923 [43,44], induline 3B (**10**) was little studied until 2012, when a study by Roy et al. revisited its chemistry to describe various N-aryl substituted analogues using a similar approach (self-condensation of aniline derivatives) [43].


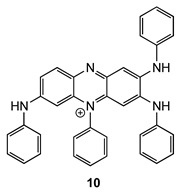


**Scheme 2:**
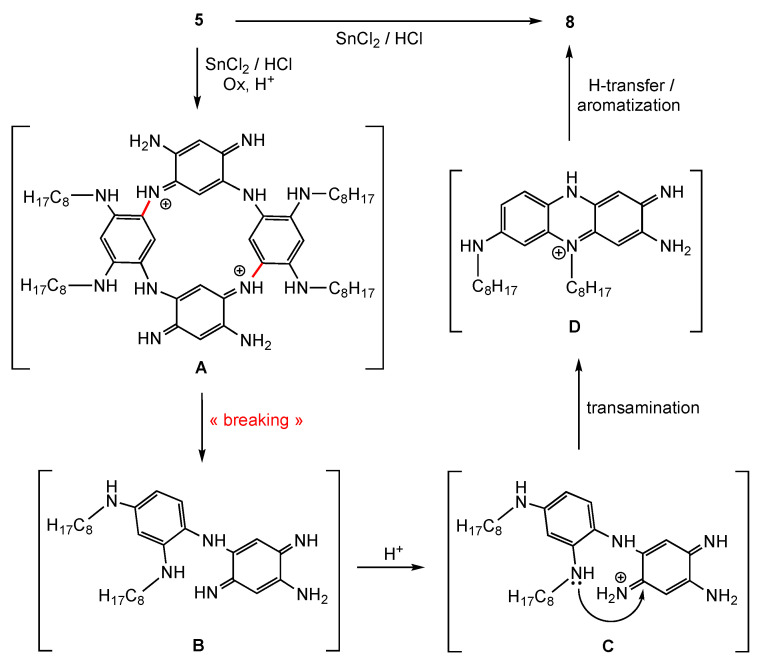
Suggested mechanism to convert **5** into **8**.

When we started to screen reaction conditions with the hope of finding an experimental procedure for the hydrogenation of azacalixarene **6**, we first avoided the use of acids and used hydrogenation on Pd/C and direct air-oxidation which led to molecule **7**. Previous works on macrocyclic chemistry reported that in the presence of acids, possible competition between degradation and hydrogenation might occur [46]. Here, we screened conditions for hydrogenation in acidic medium and we indeed observed a competing ring-opening reaction. The mechanism by which the macrocycle **5** breaks up is difficult to determine but it is wise to hypothesize a breakup within the corresponding azacalixquinarene **A** bearing amino/iminium bridges to form intermediate **B**. The protonation of this latter would afford **C**, which can then undergo a transamination reaction to form a tricyclic intermediate **D**, which will aromatize to **8** by proton migration (Scheme 2).

**Scheme 3:**
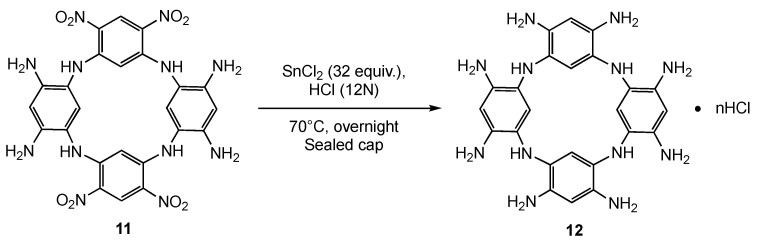
Synthesis of **12•**nHCl.

To investigate the key role of the oxidizing agent and of the N-substituents, we considered the same reaction from the unsubstituted analogue **11** [38] in the absence of air. Remarkably, its reduction using SnCl_2_·2H_2_O (32 equiv.) in the presence of HCl (12N) (same conditions as for **5**) but in a sealed tube (preventing oxidation steps) furnished the macrocycle **12•**nHCl as a yellow powder (no ring opening, Scheme 3). The degree of protonation of **12** was impossible to determine but it is reasonable to consider the protonation of at least 4 sites in order to reduce the electron density of the aromatic rings and consequently prevent oxidation of the tetraaminobenzene units.

This degree of protonation was confirmed by the ^1^H NMR proton of **12•**nHCl in D_2_O which shows only two signals at 6.31 and 7.28 ppm (see Figure S4, Supplementary Materials) in agreement with a highly symmetrical system in solution. Interestingly, when **12•**nHCl (as powder) was mixed with 10 mL of concentrated HCl and dropwise deionized water was added to dissolve all the solid under argon flux, spherical aggregates were formed after four days (see Figure S3).

**Scheme 4:**
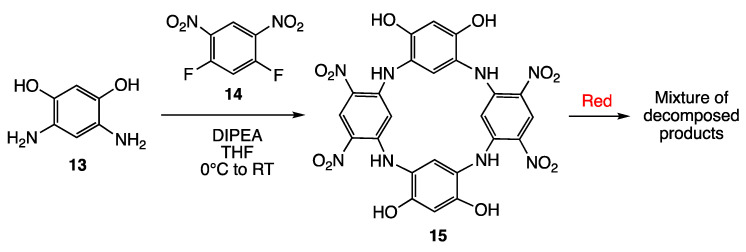
Synthesis of tetrahydroxy-tetranitroazacalixarene **15** and its reduction by using procedures similar to those for **5**.

In order to highlight the importance of the heteroatom (N vs. O) at the periphery in the reactivity of the azacalixarene, we next considered the reduction of compound **15** under the same conditions. Tetrahydroxy-tetranitro-azacalixarene **15** was first synthesized by condensation of the commercially available diaminoresorcinol **13** and difluorodinitro-benzene **14** (1 equiv.) in THF. After stirring during two days, the resulting solid in suspension was isolated by filtration affording macrocycle **15** in one step as a brown solid in a 70% yield (Scheme 4).

**Figure 4:**
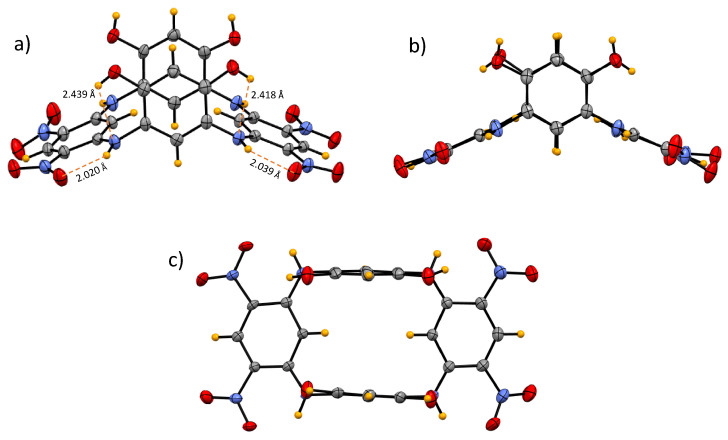
Three views of the single-crystal X-ray structure of **15**; (**a**) H-bonding interaction, (**b**) side view, (**c**) top view.

The ^1^H NMR spectrum of **15** shows an unusual high-field chemical shift of the intra-annular aromatic protons at δ 5.70 ppm that suggests a 1,3-alternate conformation, in which these protons are located inside the anisotropic shielding. This observation confirms the high stability of this specific conformation in tetranitrocalixarenes even in the presence of H donor/acceptor sites inherent to the presence of the OH groups. The structure of **15** could be fully established by X-ray analysis, which confirmed its 1,3-alternate conformation stabilized by intramolecular hydrogen bonds involving the protons of the NH bridges of the macrocycle and the NO_2_ groups, but also the participation of two hydroxy functions which interact with the nitrogen atom of the bridge (Figure 4). One of the most striking features of this structure is the sp^2^ hybridation adopted by all the nitrogen bridging atoms that are conjugated with their adjacent dinitrobenzene rings, as already observed in related tetranitro-azacalixarenes [36].

We then considered the reduction of compound **15** as for **5** as well as using many others using alternative reducing agents and/or conditions. In all cases, a full degradation of the reaction products, giving a complex mixture whose constituents could not be identified. This observation clearly confirms the key role of the amino functions at the periphery in the breaking of azacalixarenes into induline derivatives.

## 4. Materials and Methods

All reagents and solvents were purchased and used without purification. Liquid Nuclear Magnetic Resonance spectra were recorded on a Brucker advance 250 MHz (250 MHz for ^1^H NMR and 62 MHz for ^13^C) or on a JOEL Eclipse 400 MHz spectrometer. Chemical shifts are given in ppm relative to the signal (residual peak) of the solvent. The abbreviations used correspond to br = broad, s = singlet, d = doublet, t = triplet, and m = multiplet. Solid ^13^C NMR were recorded on a Bruker Avance III WB 400 spectrometer. UV-Visible spectra were recorded at room temperature with a Varian Cary 50 UV-Vis spectrophotometer. High-resolution mass spectrometry (HRMS) analysis was performed by the “Spectropole” of Aix-Marseille University. Absorptions were performed in quartz cells of 1 cm and 1 mm. Emission spectra were measured using a Horiba-Jobin Yvon Fluorolog-3 spectrofluorometer equipped with a three-slit double-grating excitation and a spectrograph emission monochromator with dispersions of 2.1 nm mm-1 (1200 grooves per mm). A 450 W xenon continuous wave lamp provided excitation. The luminescence of diluted solutions was detected at right angle using 10 mm quartz cuvettes.

Compound **8**: to a solution of compound **5** (50 mg, 0.048 mmol, 1.0 eq) in absolute EtOH (50 mL), SnCl_2_∙2H_2_O (343 mg, 1.52 mmol, 32 eq) and HCl (12N, 0.13 mL) were added. The mixture was allowed to reflux overnight, then neutralized with NaHCO_3_ before the addition of EtOH (30 mL) and water (20 mL). After concentration, the residue was extracted with DCM/EtOH (3/1, *v/v*). The red organic phase was washed with an aqueous solution of HPF_6_ (1%*w/w* in water, 4 × 150 mL) and brine (100 mL), dried with MgSO_4_ and concentrated under vacuum to afford **8** as a dark red solid (35 mg, 0.059 mmol, 62% yield). ^1^H NMR (400 MHz, CD_3_OD): δ 7.68 (d, *J* = 9.2 Hz, 1H), 7.17 (d, *J* = 8.8 Hz, 1H), 6.99 (s, 1H), 6.93 (s, 1H), 6.45 (br s, 1H), 4.53 (t, *J* = 7.6 Hz, 2H), 3.29 (m, 2H), 1.91 (quint, *J* = 7.2 Hz, 2H), 1.74 (quint, *J* = 7.2 Hz, 2H), 1.63 (quint, *J* = 7.2 Hz, 2H), 1.47–1.23 (m, 18H), 0.93–0.88 (m, 6H). ^13^C NMR (100 MHz, CD_3_OD): 154.8, 152.1, 141.1, 139.6, 136.8, 134.2, 132.8, 132.1, 121.6, 108.8, 93.7, 90.3, 44.4, 33.1, 33.1, 30.7, 30.6, 29.8, 28.5, 28.0, 27.6, 23.8, 14.5. HRMS (ESI-TOF): *m*/*z* [M + NH_4_]^+^ for C_28_H_44_N_5_^+^ calcd. 450.3591, found 450.3592, err. < 1 ppm.

Compound **12•**nHCl: precursor **11** (50 mg) and SnCl_2_ (500 mg, 32 eq) were mixed in a screw-cap vial with 10 mL of 37% HCl. The vial was then sealed with a Teflon-lined cap and placed in an oil bath at 70 °C with agitation for 20h. The clear yellow suspension was then cooled to room temperature. A volume of 40 mL of HCl (12N) was then mixed in the suspension and placed in an ultrasound bath for 10 min. The resulting solid was collected by filtration and washed successively with a mixture of MeCN and conc. HCl (40 mL/10 mL) and 2 × 20 mL of Et_2_O. The crude vanilla powder **12•**nHCl (70 mg) was dried by flux of argon and stored at −20 °C. ^1^H NMR (250 MHz, D_2_O): 7.28 (s, 4H), 6.31 (s, 4H). Solid state ^13^C NMR spectrum: 130–100 (m, sp^2^ C-C), 132–148 (m, sp^2^ C-N). Further characterization could not be carried out because of its high instability.

Compound **15**: to a solution of 1,5-difluoro-2,4-dinitrobenzene **14** (172.5 mg, 0.845 mmol, 0.9 eq) in THF (45 mL), 4,6-diaminoresorcinol dihydrochloride **13** (200 mg, 0.939 mmol, 1.0 eq) was added. The flask was sealed and degassed by 3 times of pump-argon cycling. Then, under an inert atmosphere, degassed *N*,*N*-diisopropylethylamine (DIPEA) (1.3 mL, 7.51 mmol, 8.0 eq) was added dropwise by a syringe at 0 °C. The solution was maintained at 0 °C for 6 h, and then allowed to warm at room temperature for 2 days. The resulting solid in suspension was isolated by filtration and washed with HCl (6N), EtOH and Et_2_O to afford the desired product **15** as an orange solid (177.7 mg, 0.292 mmol, 70% yield). ^1^H NMR (400 MHz, DMSO-d6): 9.71 (br s, 4H), 9.12 (br s, 4H), 9.01 (s, 2H), 6.83 (s, 2H), 6.51 (s, 2H), 5.50 (s, 2H). 13C NMR (100 MHz, DMSO-d6): 153.7, 148.9, 129.4, 128.0, 124.4, 115.8, 104.2, 94.0. HRMS (ESI-TOF): *m*/*z* [M + H]^+^ for C_24_H_17_N_8_O_12_^+^ calcd. 609.0960, found 609.0956, err. < 1 ppm.

## 5. Conclusions

We showed that depending on the reduction conditions, tetranitroazacalixarene **5** is at the crossroad of two different families of dyes including triamino-phenazinium **8** that results from a competing ring-opening reaction. The mechanism by which the macrocycle **5** breaks up is difficult to determine but we were able to highlight the key role of the amino functions at the periphery of the macrocycle and the need of an oxidizing agent (air). We are aware that the degradation of a sophisticated macrocycle (**5**) is not an efficient strategy to access induline derivatives by comparison with the straightforward synthesis recently described based on stepwise nucleophilic aromatic substitutions [41]. This work is more concerned with describing the versatility of azacalixarene-type macrocycles, beyond their “classical” use in supramolecular chemistry.

## Data Availability

Data are contained within the article and Supplementary Materials.

## Supplementary Materials

The following supporting information can be downloaded at: https://www.mdpi.com/article/10.3390/molecules28248113/s1. ^1^H and ^13^H NMR data. Crystal data for complex **15** has been deposited in the CCDC database as deposition number 2308474 and can be accessed from there. The cif file for **15** is available as supplementary information on request.
