# Supplementary material for: Breaking Azacalix[4]arenes into Induline Derivatives
Source: Molecules. 2023 Dec 15;28(24):8113. doi: 10.3390/molecules28248113 (PMC10746034; doi:10.3390/molecules28248113)
Supplement: Supplementary file 1 [file molecules-28-08113-s001.zip › molecules-2717735-supplementary.pdf]

Supplementary informations

Breaking azacalix[4]arenes into induline derivatives

Zhongrui Chen, Gabriel Canard, Olivier Grauby, Benjamin Mourot and Olivier Siri*

Centre Interdisciplinaire de Nanoscience de Marseille (CINaM), UMR 7325 CNRS Aix-Marseille Université, Campus de Luminy, case 913 F-13288 Marseille cedex 09 (France)

* Correspondence: olivier.siri@univ-amu.fr

1. <u>NMR spectra</u>

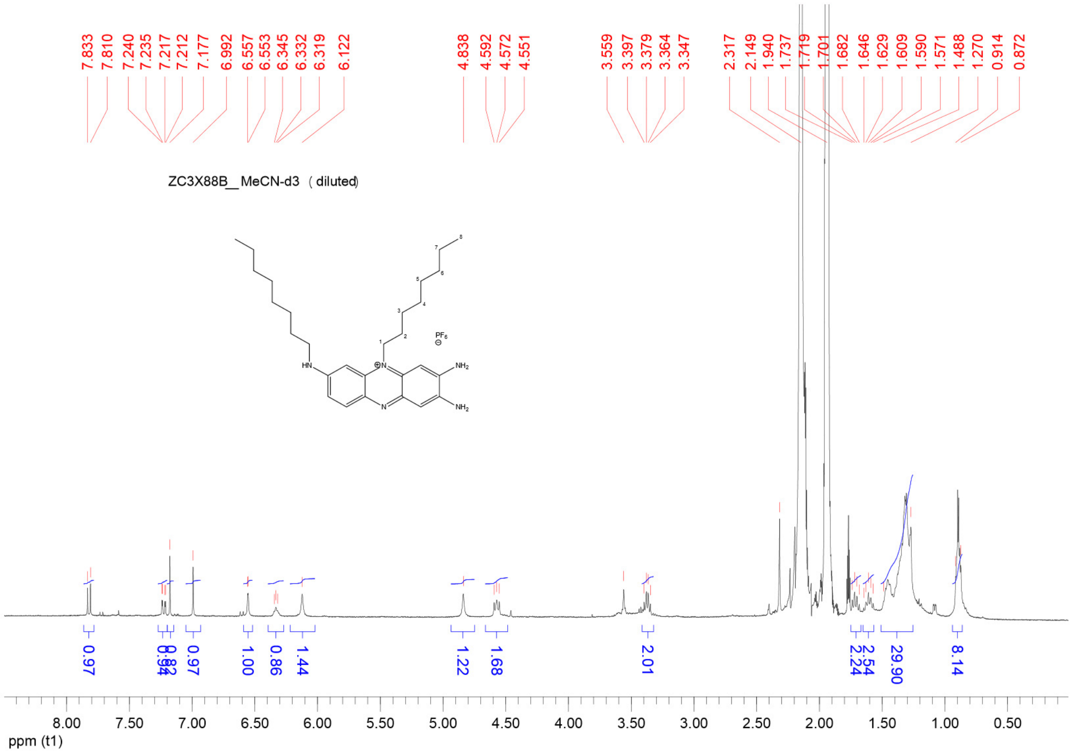

Figure S1. ^1H NMR spectrum of **8** in CD_3CN

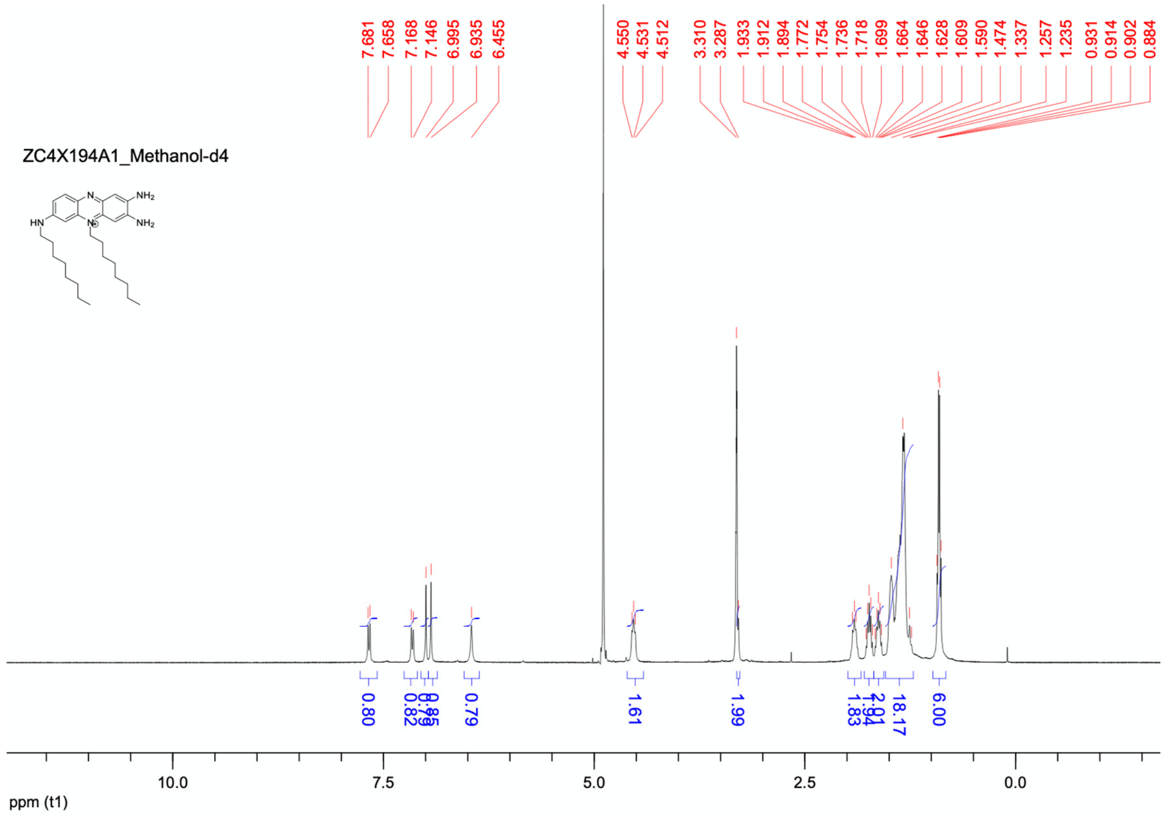

Figure S2. ^{1}H NMR spectrum of **8** in MeOD

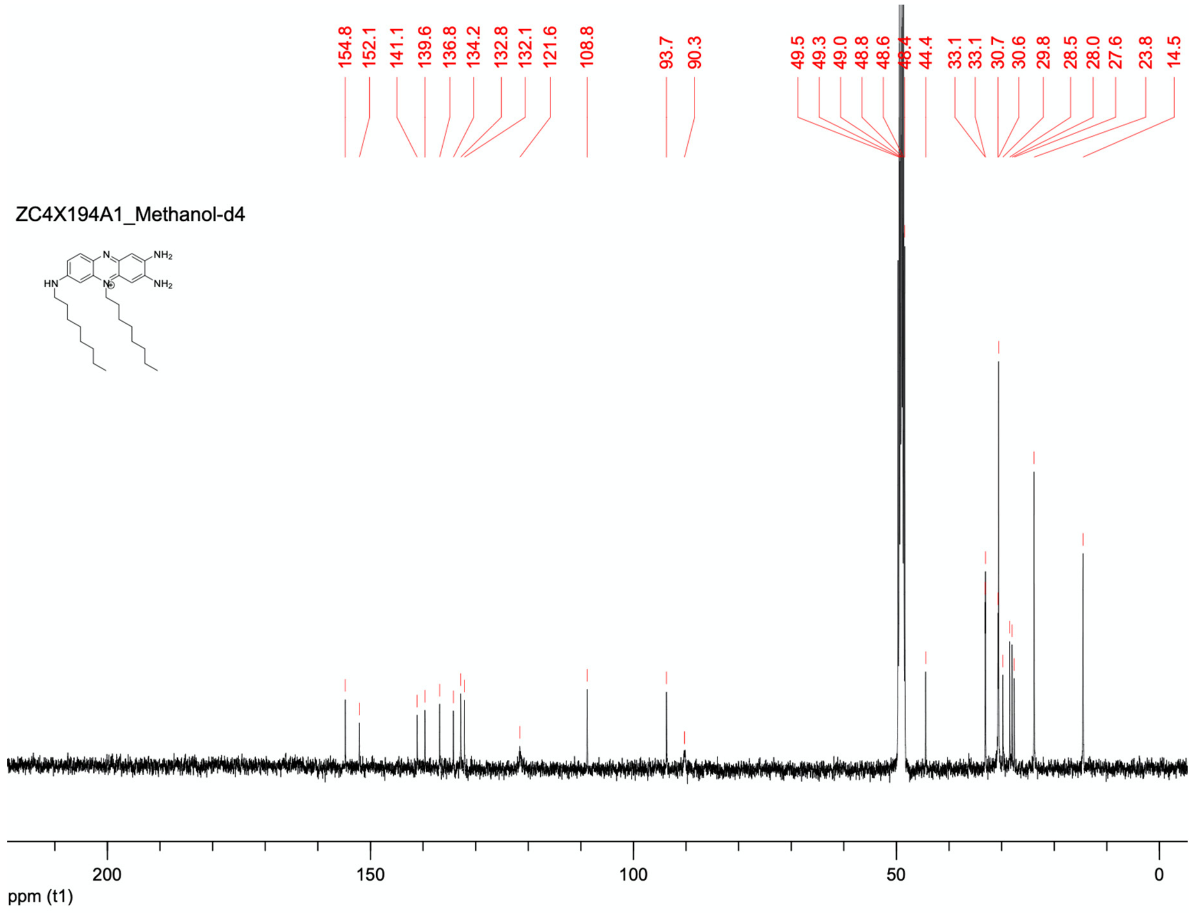

Figure S3. ^{13}C NMR spectrum of **8** in MeOD

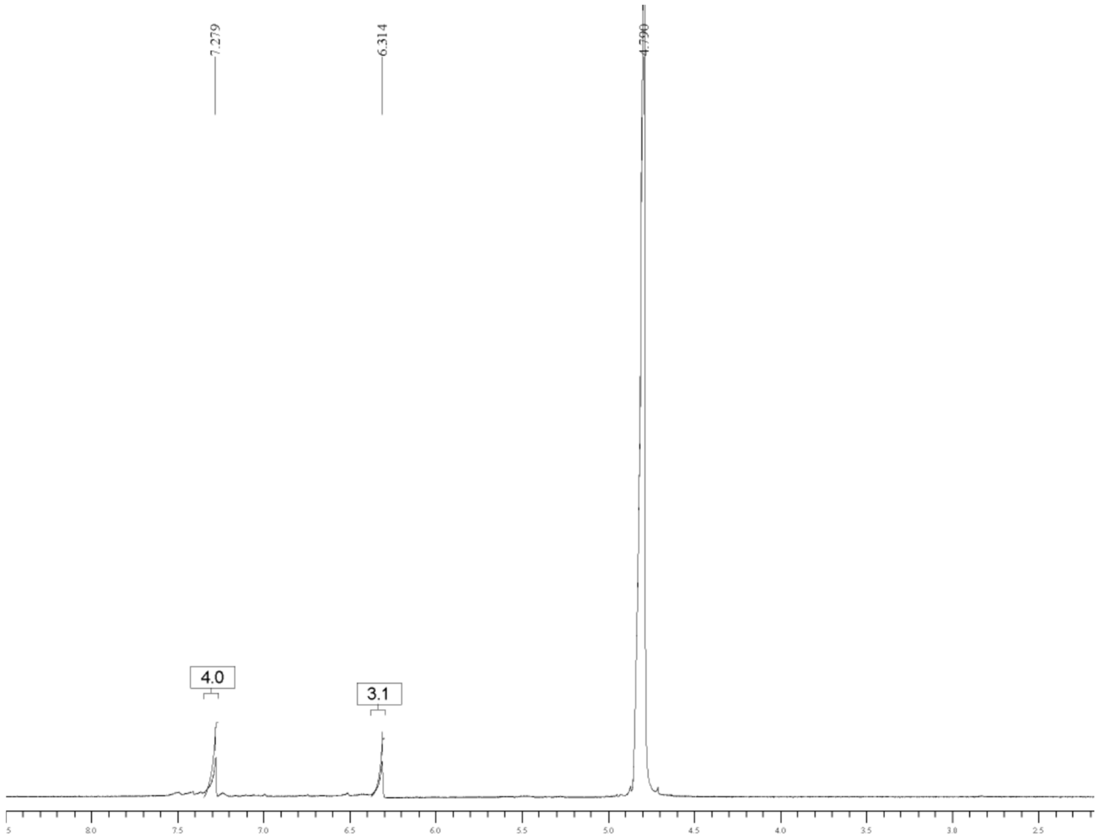

Figure S4. ^{1}H spectrum of **12•**nHCl in D_2O

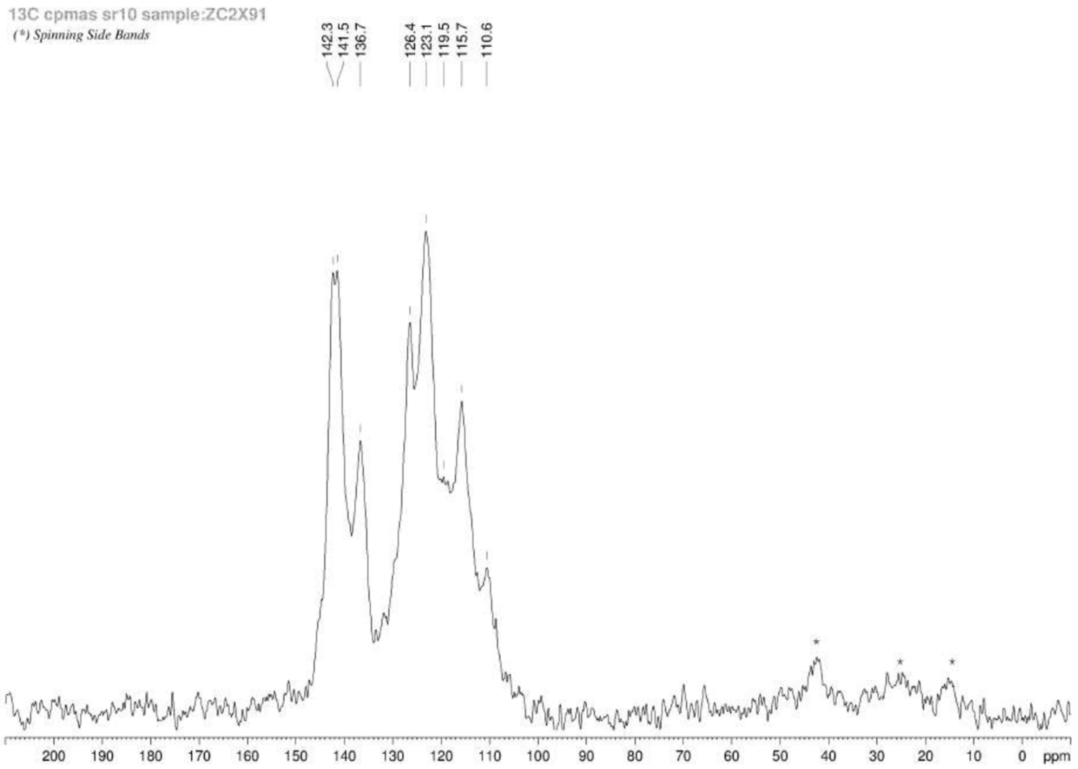

Figure S5. solid state ^{13}C NMR spectrum of **12•**nHCl

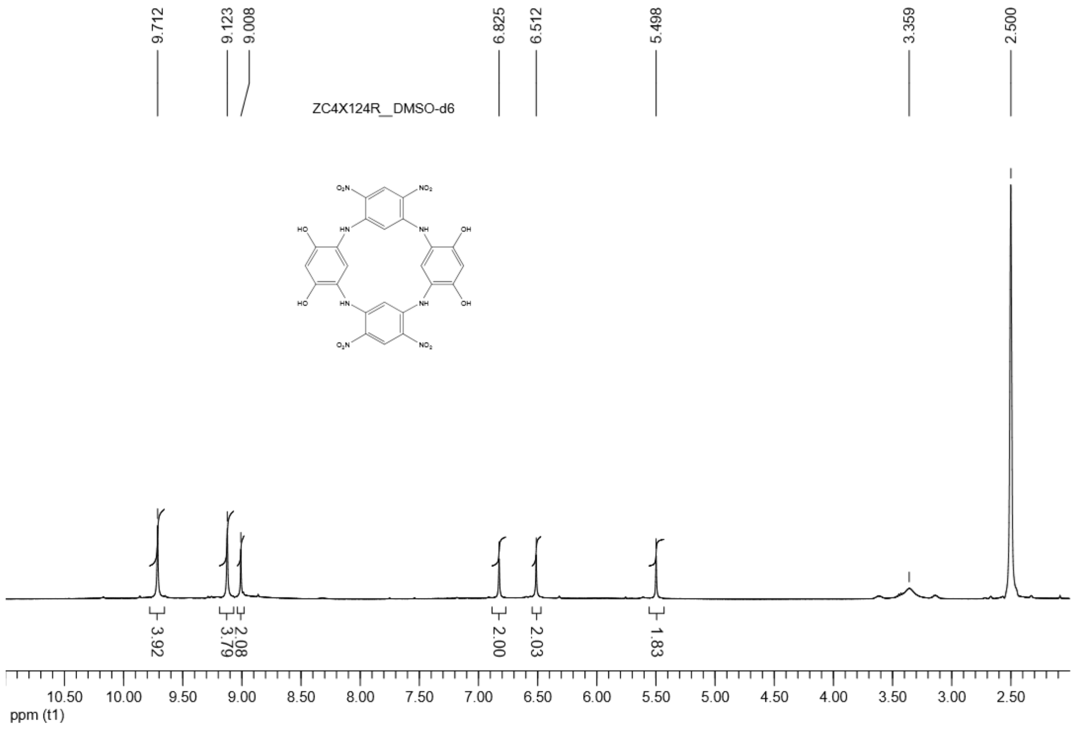

Figure S6. ^{1}H NMR spectrum of **15** in DMSO

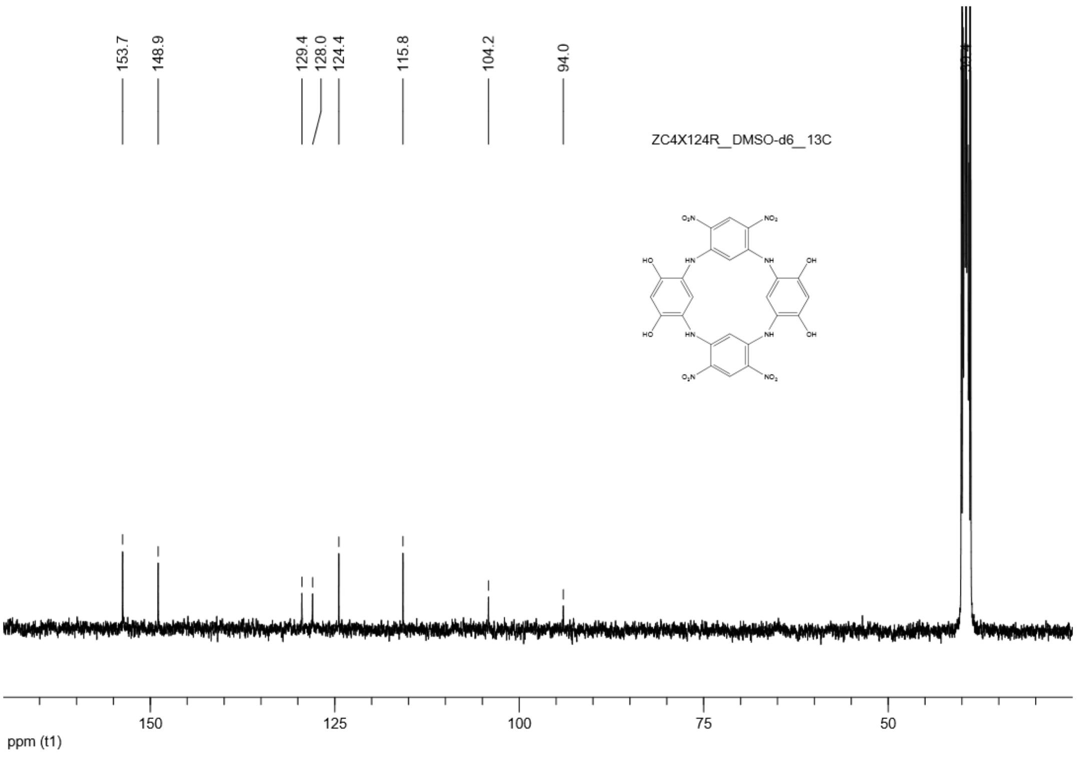

Figure S7. ^{13}C NMR spectrum of **15** in DMSO

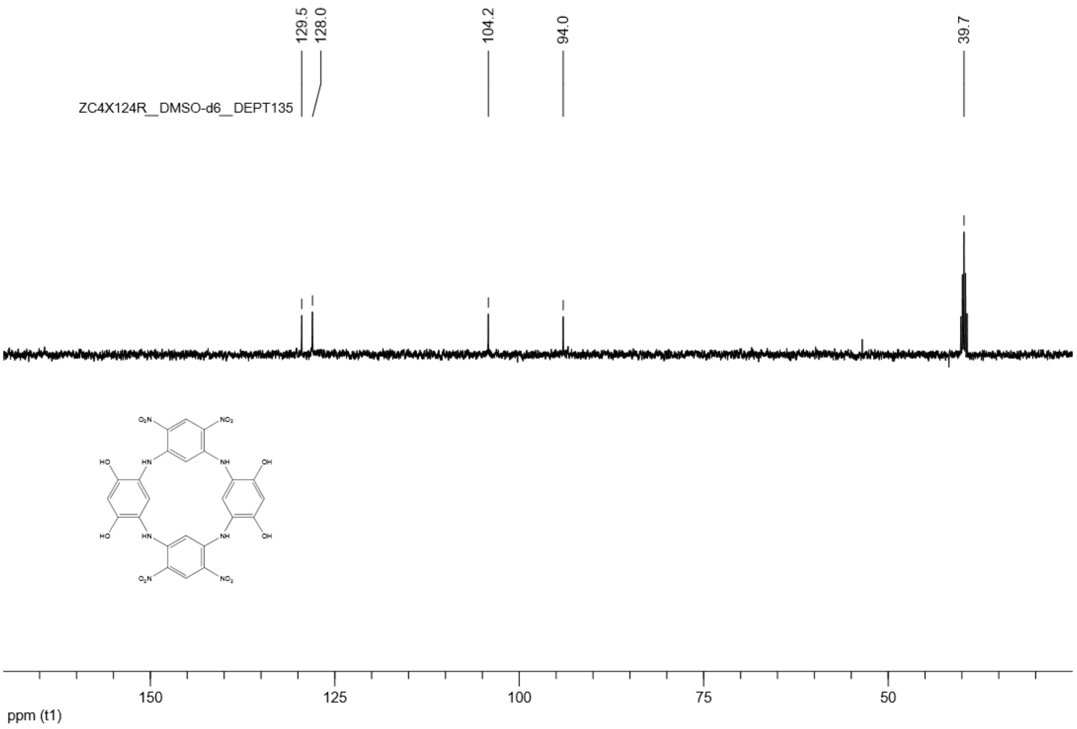

Figure S8. ^{13}C NMR spectrum of **15** (DEPT) in DMSO

2. <u>X-ray Structure of molecule **15**</u>

Alert level B in checkcif:

```
Alert level B
              Crystal system given = triclinic
PLAT430_ALERT_2_B Short Inter D...A Contact  S1B
O10      ..        2.82 Ang.
```

Comment on this alert:

If we look at the structure by extending the molecule a little towards its neighbors, we see that the NO_2 bearing the oxygen O(10) is the only one of the 4 out of the plane.

What's more, the DMSO involved in the interaction defined as short by the check-cif is disordered on 2 sites, with the S(1B) sulfur on one of the two sites interacting with O(10) on this NO_2. The two observations combined show quite clearly that there is a particular interaction at this level.

Hence the short distance observed by the check-cif.

3. <u>SEM analysis</u>

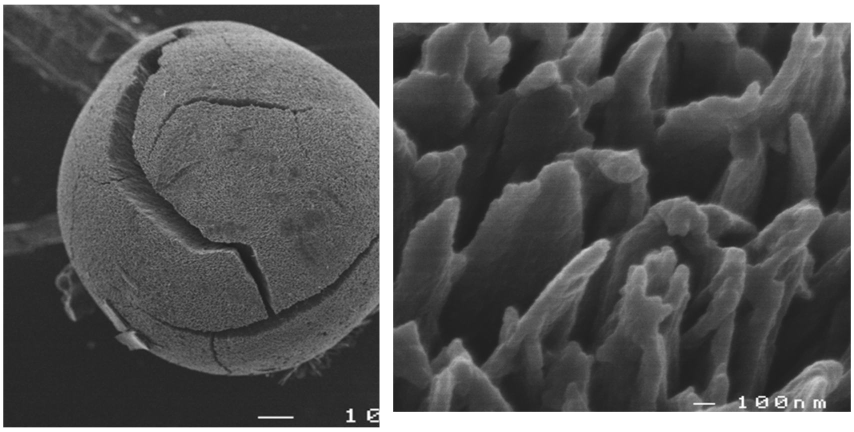

Figure S9. SEM images of **12•**nHCl-aggregates.
